# Development of a new clinical index to easily assess frailty of elderly patients with multiple myeloma in Asian population

**DOI:** 10.1038/s41598-021-02433-6

**Published:** 2021-11-25

**Authors:** Ho Sup Lee, JiHyun Lee, Jae-Cheol Jo, Sung-Hoon Jung, Je-Jung Lee, Dajung Kim, Sangjin Lee, Kevin Song

**Affiliations:** 1 Department of Internal Medicine, Kosin University College of Medicine, Busan, South Korea; 2 Department of Internal Medicine, Dong-A University College of Medicine, Busan, South Korea; 3 Department of Hematology and Oncology, Ulsan University Hospital, University of Ulsan College of Medicine, Ulsan, South Korea; 4 Department of Hematology-Oncology, Chonnam National University Hwasun Hospital, Hwasun, Jeollanamdo South Korea; 5 Department of Statistics, Pusan National University, Busan, South Korea; 6 Division of Hematology, Leukemia/Bone Marrow Transplant Program of British Columbia, Vancouver General Hospital, BC Cancer, 2775 Laurel St, 10th Fl, Vancouver, BC V5Z1M9 Canada

**Keywords:** Risk factors, Oncology, Cancer

## Abstract

The number of elderly people is rapidly growing, and the proportion of elderly patients with multiple myeloma (MM) continues to increase. This study aimed to develop a frailty assessment tool based on clinical data and to estimate its feasibility in elderly patients with MM. This study analyzed data from 728 elderly transplant-ineligible patients with newly diagnosed MM who were treated between January 2010 and October 2019. Our clinical frailty index included age (< 75, and ≥ 75 years), Charlson comorbidity index (CCI; < 3 and ≥ 3), and Eastern Cooperative Oncology Group performance status score (ECOG score; 0, 1–2, and ≥ 3). Patients were classified as fit, intermediate, or frail if they had a score of 0, 1, or ≥ 2, respectively. The overall survival rates differed significantly according to frailty (fit vs. intermediate: hazard ratio [HR] = 2.41; 95% confidence interval [CI] = 1.43–4.06; *P* = 0.001; fit vs. frail: HR = 4.61; 95% CI = 2.74–7.77; *P* < 0.001 and intermediate vs. frail: HR = 1.91, 95% CI = 1.49–2.45, *P* < 0.001, respectively). The frail had significantly shorter EFS compared with the fit and intermediate group in our frailty index (fit vs. intermediate: HR = 1.34, 95% CI = 0.92–1.96, *P* = 0.132; fit vs. frail: HR = 2.06, 95% CI = 1.40–3.02, *P* < 0.001; and intermediate vs. frail: HR = 1.53, 95% CI = 1.22–1.92, *P* < 0.001, respectively). The new clinical frailty index, which is based on age, CCI, and ECOG PS, can easily assess frailty in elderly patients with MM and can be helpful in predicting survival outcomes in real world clinical setting.

## Introduction

Globally, the number of elderly people is rapidly growing, and the proportion of elderly patients with multiple myeloma (MM) continues to increase^1,2^. Approximately one-third of patients are > 75 years old at diagnosis^3^. Moreover, at least 30% are frail, both due to decline in physical capacity, presence of comorbidities, polypharmacy, nutritional status, and cognitive impairment^4^. Until now, data on the efficacy and feasibility of current standards of care in frail patients are lacking. Many clinical trials for elderly patients have largely been based on fit rather than frail patients who are excluded due to the presence of comorbidities, abnormal laboratory test results, and physical disability^5,6^.

With the increasing interest in elderly frail patients, several frailty assessment tools have been developed^7–9^. Currently, the commonly used frailty models include the International Myeloma Working Group (IMWG) frailty score and revised myeloma comorbidity index (R-MCI)^7,8^. Engelhardt et al.^8^, conducted a comprehensive comorbidity, frailty, and disability evaluation in 801 consecutive patients with myeloma. Renal and lung impairments, Karnofsky performance status, frailty, and age were determined as significant risk factors affecting overall survival (OS). These were combined in a weighted R-MCI, allowing for the identification of low (≤ 3 [n = 247, 30.8%]), intermediate (4–6 [n = 446, 55.7%]), and high-risk patients (> 6 [n = 108, 13.5%]). However, the R-MCI is a complex tool and may be inconvenient for clinical use because seven variables, including cytogenetics, are required. Approximately 10% of elderly patients are classified as being at high risk according to the R-MCI. In a pooled analysis of 869 patients with newly diagnosed MM, Palumbo et al.^7^, demonstrated that their IMWG score defined fit, intermediate-fit, and frail patients and predicted the risk of mortality. The IMWG score combines age, activities of daily living (ADLs), instrumental activities of daily living (IADLs), and Charlson comorbidity index (CCI). However, the inclusion of ADLs and IADLs may not be straightforward in clinical practice. Therefore, Facon et al. determined outcomes based on frailty using scores for age, CCI, and Eastern Cooperative Oncology Group performance status (ECOG PS) without ADLs and IADLs^10^. The simplified frailty scale showed that frail patients had worse progression-free and OS than non-frail patients. However, > 40% of patients are considered frail using this tool. In addition, this study is an analysis of patients who have been enrolled in a prospective study which had exclusion criteria.

There is an unmet need for a frailty assessment tool that is easy to use for elderly frail patients with MM in the real world clinical setting. If frailty can be estimated using an accurate frailty assessment tool, it would be helpful when treating frail patients, improve survival, and reduce toxicity. The primary purpose of this study was to develop a new simple and useful frailty index. Therefore, this study aimed to develop a new clinical frailty index and to estimate its feasibility based on readily available and routinely collected clinical data that could predict OS in elderly transplant-ineligible patients with MM in a real world setting.

## Patients and methods

### Patients population

The data of this study were collected from the medical charts of 728 elderly patients with newly diagnosed MM who were treated by specialized physicians at academic institutions in South Korea between January 2010 and October 2019. This study included patients who had been diagnosed with MM by standard criteria and who underwent initial treatment determined by physicians and had not undergone transplantation. Cytogenetic risk was determined using conventional cytogenetics or fluorescence in situ hybridization. Factors indicative of high risk included t(4;14), 17p deletion, t(14;16), t(14;20), gain(1q), del(13), and non-hyperdiploidy, whereas factors indicative of standard risk included t(11;14), t(6;14), and all the others^11^. The revised international staging system (R-ISS) is based on the international staging system (ISS) stage, cytogenetic risk, and serum lactate dehydrogenase (LDH) levels at diagnosis^12^. The most commonly used initial treatment was bortezomib based chemotherapy because the bortezomib, melphalan, and prednisolone regimen has been reimbursed by the Health Care Insurance System of South Korea since 2010, whereas lenalidomide was reimbursed only from December 2017. This study was approved by the institutional review board of Kosin University Gospel Hospital.

### Design and assessment of new frailty index scoring system

The new clinical frailty index was designed based on clinical experience including only three clinical factors: age, CCI, and ECOG PS (Table 2). The risk index was based upon the log of the hazard ratio which were independent prognostic factors in univariate and multivariate analyses of OS (Table 3). Age was scored 0 or 1 if the patient was < 75 or ≥ 75 years old, respectively; CCI was scored 0 or 1 if it was < 3 or ≥ 3, respectively; and ECOG PS was scored 0, 1, or 2 if it was 0, 1–2, or ≥ 3, respectively. Patients were classified as fit, intermediate, or frail if they had a total score of 0, 1, or ≥ 2, respectively. The differences in survival outcomes were analyzed according to frailty as assessed using our frailty index, the R-MCI, and the simplified frailty scale. We also assessed the differences in early mortality and cause of death according to three different frailty models. The simplified frailty scale was used in this study instead of the IMWG frailty index because our study did not record ADL/IADL data^7,13^. Patients were divided into frailty categories using baseline patient characteristics, including age, CCI, and ECOG PS. The R-MCI was calculated based on the presence of impaired lung and kidney function, Karnofsky performance status, frailty, age, and unfavorable cytogenetics^8^. The three prognostic risk groups were classified as low-, intermediate-, and high-risk.

### Statistical analysis

Patient demographic and clinical data are described using numerical epidemiological/statistical methods. The percentage of patients who presented with objective responses, as well as the percentage of patients who experienced adverse events, were examined using parametric and non-parametric tests. We developed our frailty index and assessed its feasibility based on our data set. Moreover, we compared our frailty index with the R-MCI and the simplified frailty scale. Event-free survival (EFS) was measured from the date of treatment initiation until progression, discontinuation due to toxicity, or death from any cause. OS was measured from the date of diagnosis until death from any cause. OS was censored at the last date of patient follow-up. Early mortality rates were defined as death due to any cause within 90 days after treatment initiation. Death due to toxicity was defined as mortality due to chemotherapy-induced toxicity without disease progression. EFS and OS were analyzed using the Kaplan–Meier methodology, and a log-rank test was used for comparisons between different frailty categories. Various patient characteristics were compared using the chi-square test or Fisher’s exact test for categorical variables and the Mann–Whitney U test for continuous variables. Multivariate analysis was performed to identify independent prognostic factors for EFS and OS in frail patients. Following univariate analysis, variables with *P* < 0.05 or relevant clinical factors were analyzed once more using multivariate analysis. The Cox proportional hazard regression model was used for multivariate analysis of independent prognostic factors of survival. There were missing values for some cytogenetics and laboratory data, including β_2_ microglobulin (β_2_MG) and LDH values. Those with missing variables were excluded from the statistical analysis of the specific variable. Statistical analysis was performed using SPSS version 26 software (IBM, Armonk, NY, USA).

### Compliance with ethical standards

All procedures performed in studies involving human participants were in accordance with the ethical standards of the institutional and national research committee and with the Declaration of Helsinki (1964) and its later amendments or comparable ethical standards. According to the Declaration of Helsinki, the trial was approved by the Institutional Research Ethics Board of Kosin University Gospel Hospital, which waived the requirement for informed consent given the retrospective nature of this study (IRB approval number 2020-01-005).

## Results

### Patients’ characteristics

The demographic and disease characteristics of the 728 participants are shown in Table 1. The median age of patients was 70.7 years (range, 60–91 years). 273 (37.4%) patients had an ECOG PS score of ≥ 2 and 248 (34.0%) patients had a CCI score of ≥ 2 in our data set, respectively. The initial treatment regimens showed mainly bortezomib based chemotherapy in our data set. About 76.1% of patients received bortezomib based chemotherapy, 8.5% received immune modulatory drugs (IMiDs) based chemotherapy, and 9.2% received other treatments, including melphalan, respectively. The proportion of patients with renal impairment (creatinine clearance < 60 mL/min) was 11.3%, high-risk R-ISS (stage III) was 27.1% and abnormal LDH levels was 20.3%. The median β_2_MG was 4.6 mg/L.

**Table 1:** Patients characteristics.

Variable (n = 728)		N (%)
Age, years	Median (range)	70.7 (60–91)
Sex	Male	384 (52.7)
	Female	344 (47.3)
ECOG PS	0	144 (19.8)
	1	311 (42.7)
	2	199 (27.3)
	3	70 (9.6)
	4	4 (0.5)
CCI	0	258 (35.4)
	1	222 (30.5)
	2	140 (19.2)
	≥ 3	108 (14.8)
β_2_MG, mg/L	Median (range)	4.6 (0.3–67.4)
	NA	29
ClCr, ml/min	≥ 60	641 (88.0)
	< 60	82 (11.3)
	NA	5 (0.7)
LDH	Normal	440 (60.4)
	Abnormal	148 (20.3)
	NA	140 (19.3)
Cytogenetic risk	Favorable	254 (34.9)
	Unfavorable	137 (18.8)
	NA	337 (46.3)
R-ISS	I	40 (5.5)
	II	306 (42.0)
	III	197 (27.1)
	NA	185 (30.9)
Initial treatment	Bortezomib combined	554 (76.1)
	IMiDs combined	62 (8.5)
	Others	67 (9.2)
	NA	45 (6.2)

*ECOG PS*, Eastern Cooperative Oncology Group performance status; *CCI*, Charlson comorbidity index; *β*_*2*_*MG*, β_2_ microglobulin; *ClCr*, creatinine clearance; *LDH*, lactate dehydrogenase; *R-ISS*, revised international staging system; *NA*, not assessed; *IMiDs*, immunomodulatory drugs.

### Development of a new clinical frailty index based on age, CCI, and ECOG PS

The results of the univariate and multivariate analyses are shown in Table 3. In the univariate analysis, the median OS was shown to be significantly different between age groups (< 75, and ≥ 75 years: hazard ratio [HR] = 1.65, 95% confidence interval [CI] = 1.26–2.17; *P* < 0.001), CCI (< 3 vs. ≥ 3: HR = 2.55, 95% CI = 1.92–3.39, *P* < 0.001), ECOG PS score (0 vs. 1–2, and 0 vs. ≥ 3: HR = 2.16, 95% CI = 1.44–3.23; *P* < 0.001 and HR = 3.72, 95% CI = 2.25–6.14; *P* < 0.001, respectively), β_2_MG (< 5.5 and ≥ 5.5 mg/L: HR = 1.86, 95% CI = 1.45–2.39; *P* < 0.001) and LDH (normal vs. abnormal: HR = 1.82, 95% CI = 1.36–2.43; *P* < 0.001) levels, and R-ISS (stage I vs. II and I vs. III: HR = 1.50, 95% CI = 0.76–3.00; *P* = 0.246 and HR = 2.83, 95% CI = 1.42–5.63; *P* < 0.001, respectively). The multivariate analysis revealed that the independent prognostic factors for OS were age (< 75, and ≥ 75 years: HR = 1.79, 95% CI = 1.28–2.51; *P* = 0.001), CCI (< 3 vs. ≥ 3: HR = 2.89, 95% CI = 1.96–4.27, *P* < 0.001), ECOG PS score (0 vs. 1–2, and 0 vs. ≥ 3: HR = 2.08, 95% CI = 1.19–3.64; *P* = 0.010 and HR = 3.18, 95% CI = 1.62–6.25; *P* = 0.001, respectively), LDH (normal vs. abnormal: HR = 1.52, 95% CI = 1.06–2.19; *P* = 0.024) levels and β_2_MG (< 5.5 and ≥ 5.5 mg/L: HR = 1.65, 95% CI = 1.15–2.37; *P* = 0.006) level.

### Feasibility assessment of new clinical frailty index

**Figure 1:**
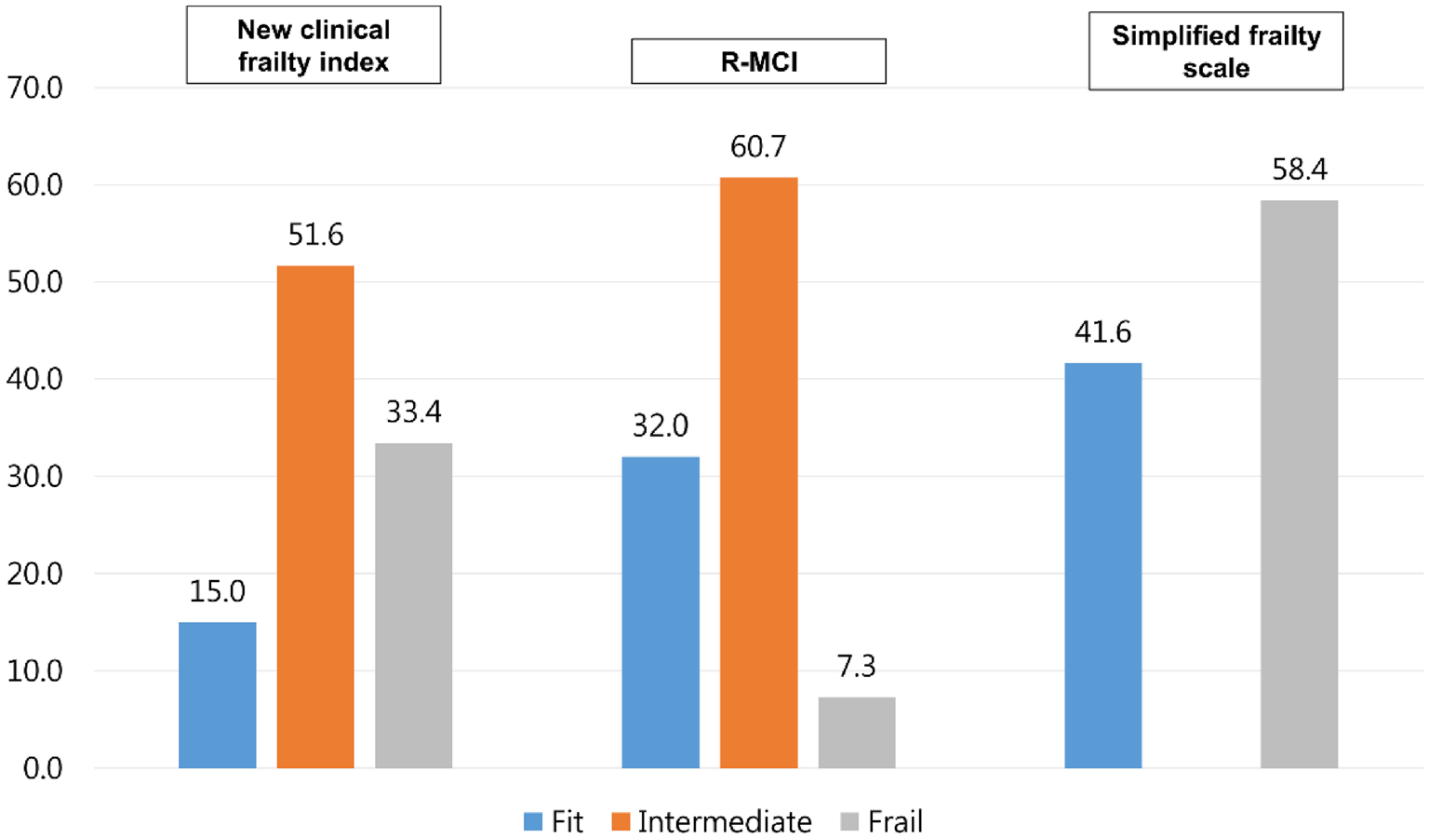
Proportion of patients classified in each frailty group according to the frailty model.

The differences in proportions of the classified frailty groups based on the three different frailty assessment tools are shown in Fig. 1. The respective proportion of patients classified as fit, intermediate, and frail according to our frailty index was 15.0%, 51.6%, and 33.4% in our data set, respectively. However, the proportion of patients classified as low, intermediate, and high risk based on the R-MCI was 32.0%, 60.7%, and 7.3% and the proportion of patients classified as non-frail or frail according to the simplified frailty scale was 41.6% and 58.4% in our data set, respectively.

**Figure 2:**
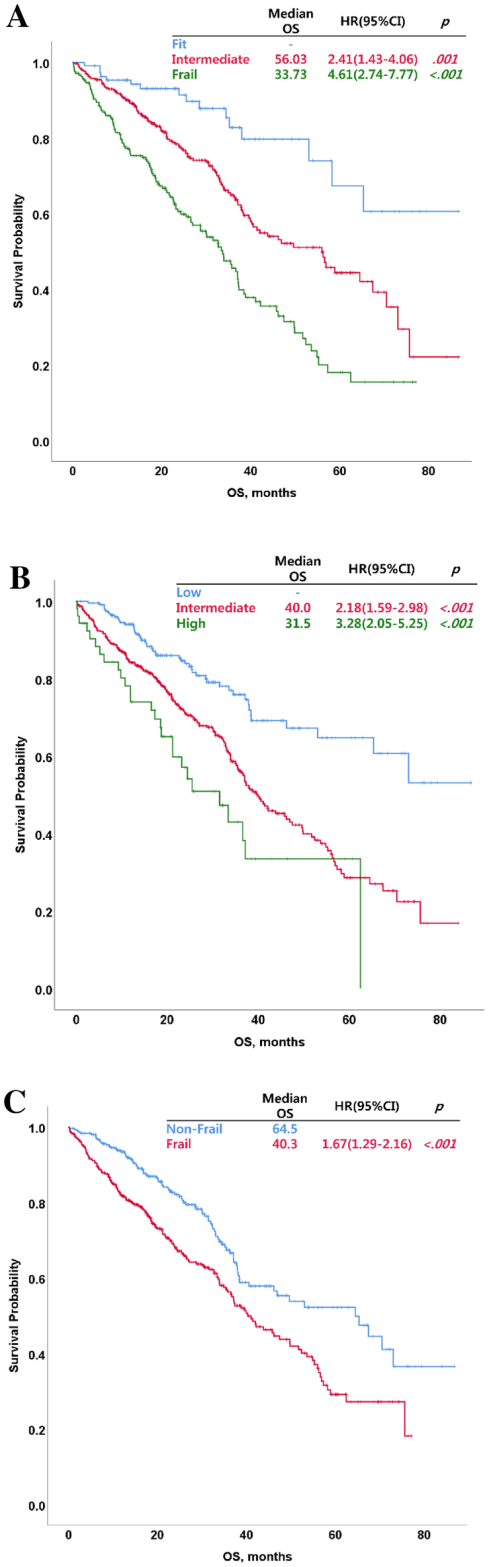
Overall survival (OS) by group as identified using different frailty assessment tools. OS of groups classified using the new clinical frailty index (**A**), revised myeloma comorbidity index (**B**), and simplified frailty scale (**C**).

**Figure 3:**
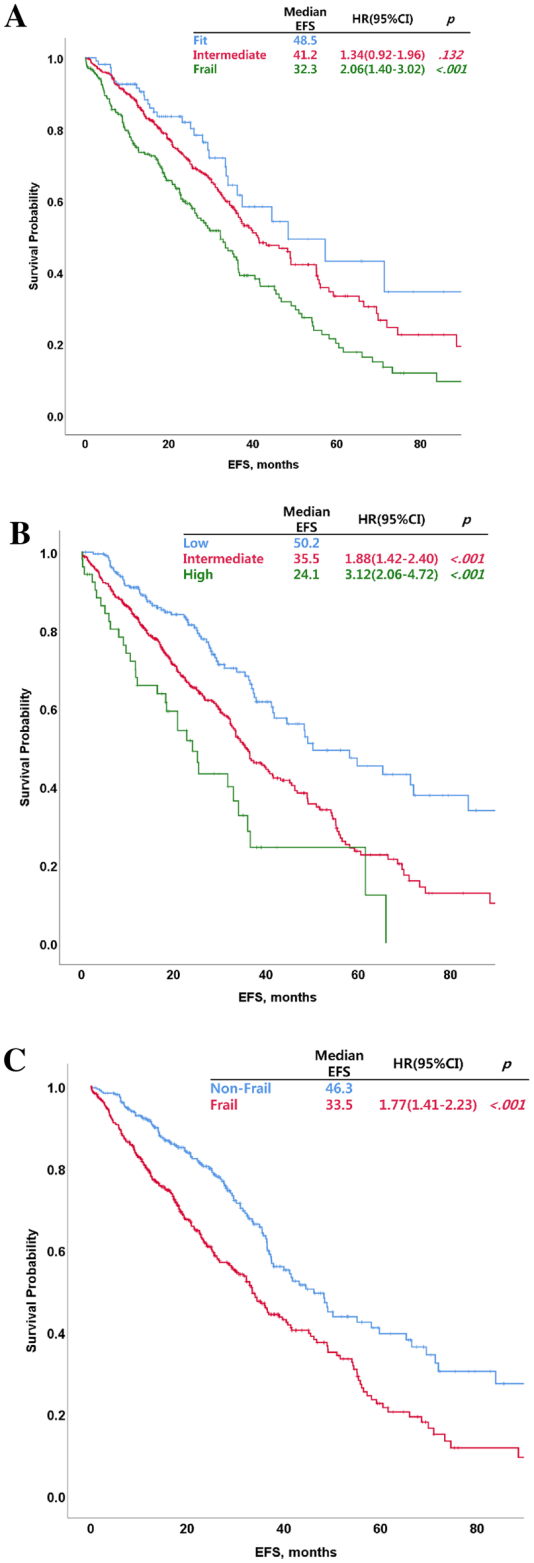
Event-free survival (EFS) by group as identified using different frailty assessment tools. EFS of groups classified using the new clinical frailty index (**A**), revised myeloma comorbidity index (**B**), and simplified frailty scale (**C**).

The differences in OS and EFS according to frailty as defined by the three frailty models were analyzed (Table 4). The intermediate and frail had significantly shorter OS compared with the fit in our frailty index (fit vs. intermediate: hazard ratio [HR] = 2.41; 95% confidence interval [CI] = 1.43–4.06; *P* = 0.001; fit vs. frail: HR = 4.61; 95% CI = 2.74–7.77; *P* < 0.001 and intermediate vs. frail: HR = 1.91, 95% CI = 1.49–2.45, *P* < 0.001, respectively, Fig. 2A). The intermediate and high in the R-MCI model and the frail in the simplified frailty scale also had significantly shorter OS compared with the low in the R-MCI and the non-frail in the simplified frailty scale (R-MCI, low vs. intermediate: HR = 2.18, 95% CI = 1.59–2.98, *P* < 0.001; low vs. high: HR = 3.28, 95% CI = 2.05–5.25, *P* < 0.001, and intermediate vs. high: HR = 1.51, 95% CI = 1.01–2.27, *P* = 0.045, respectively, Fig. 2B; and simplified frailty scale, non-frail vs. frail: HR = 1.67, 95% CI = 1.29–2.16; *P* < 0.001, Fig. 2C). The frail had significantly shorter EFS compared with the fit group in our frailty index (fit vs. intermediate: HR = 1.34, 95% CI = 0.92–1.96, *P* = 0.132; fit vs. frail: HR = 2.06, 95% CI = 1.40–3.02, *P* < 0.001; and intermediate vs. frail: HR = 1.53, 95% CI = 1.22–1.92, *P* < 0.001, respectively, Fig. 3A). The intermediate and high in the R-MCI model and the frail in the simplified frailty scale also had significantly shorter EFS compared with the low in the R-MCI and the non-frail in the simplified frailty scale (R-MCI, low vs. intermediate: HR = 1.88, 95% CI = 1.42–2.40, *P* < 0.001; low vs. high: HR = 3.12, 95% CI = 2.06–4.72, *P* < 0.001, and intermediate vs. high: HR = 1.70, 95% CI = 1.18–2.45, *P* = 0.005, respectively, Fig. 3B; simplified frailty scale, non-frail vs. frail: HR = 1.77, 95% CI = 1.41–2.23, *P* < 0.001, Fig. 3C).

## Discussion

In this study, we demonstrate a newly developed frailty index in a large number of patients who were relatively uniformly treated with 76% having received bortezomib based therapy as initial treatment. This frailty index is based on age, CCI, and ECOG PS that can be easily used to classify frail patients and predict survival outcomes in clinical practice. Age, CCI, and ECOG PS have been regarded as important prognostic factors and useful factors for developing frailty models^7–9,14–16^. Our frailty index is similar to the simplified frailty scale described by Facon et al.^13^, which also used these factors. Patients were divided into frailty categories using baseline characteristics, including age (< 76, 76–80, and > 80 years), CCI (≤ 1 and > 1), and ECOG PS score (0, 1, and 2) instead of the EuroQol five-dimensional (EQ-5D) questionnaire^13^. Each category was scored with a value of 0, 1, or 2, respectively. However, patients were classified in the frail group using this model because those aged > 80 years or with ECOG PS score of 2 were classified as frail. In this study, we focused on developing a frailty model that can easily classify frail patients. Our frailty index has a different categorization and scoring system from that of the simplified frailty scale (Table 2). Our frailty index uses a different scoring system based on clinical experience. Age (< 75 vs. ≥ 75 years), CCI (< 3 vs. ≥ 3), and ECOG (0 vs. 1–2 vs. ≥ 3) were independent prognostic factors in univariate and multivariate analyses of OS (Table 3). However, R-ISS was not shown independent prognostic factor in this study. Fluorescence in situ hybridization (FISH) and cytogenetic tests were conducted in only about 50% of patients due to cost and patient refusal. In addition, some FISH results may have inaccuracies in some of the patients for methodological reasons as they were done a number of years ago. This may have contributed to the lack of significance of the R-ISS. With the increasing interest in elderly patients with MM, appropriate frailty classification is important to physicians who treat frail elderly patients^4,5,17^. Several frailty assessment tools have been developed and validated^18^. The R-MCI, IMWG frailty scale, and simplified frailty scale have been used to classify frail patients with MM^7,8,13^. The IMWG frailty score is based on age, comorbidities, and cognitive and physical conditions and was developed to identify three groups: fit (score = 0, 39%), intermediate fitness (score = 1, 31%), and frail (score ≥ 2, 30%)^7^. The IMWG frailty score predicts mortality and risk of toxicity in elderly patients with myeloma. However, this assessment tool requires the use of questionnaires examining factors such as ADLs and IADLs. R-MCI is determined based on renal and lung impairments, Karnofsky performance status, frailty, and age as significant factors affecting OS^8^. These were combined in a weighted R-MCI, allowing for the identification of fit (≤ 3 [n = 247, 30.8%]), intermediate-fit (4–6 [n = 446, 55.7%]), and frail patients (> 6 [n = 108, 13.5%]). The R-MCI includes an accurate assessment of patients’ physical conditions. However, seven clinical factors are needed to assess frail patients, including cytogenetics, which cannot be performed in elderly patients with low socioeconomic status. Recently, Cook et al. generated a more trajectory-based objective risk score incorporating age, ECOG PS, C-reactive protein (CRP), and ISS, which was able to discriminate not only therapy-related toxicity and regimen completion, but also survivorship and effect on quality of life^9^. We did not use this model for comparison, because we did not have CRP as a part of our dataset and the UK model is relatively less commonly used than the IMWG and R-MCI models. The simplified frailty scale was investigated to determine outcomes based on frailty using scores for age, CCI, and ECOG PS, instead of the EQ-5D QOL questionnaire. Frail patients (49%) have worse progression-free and OS than non-frail patients (51%)^13^. Although this tool can be easily replicated and may help classify frail and non-frail patients, too many patients are easily classified into the frail group.

**Table 2:** Newly developed frailty scale scoring system.

Category	Score
**Age, years**	
< 75	0
≥ 75	1
**Charlson comorbidity index score**	
< 3	0
≥ 3	1
**ECOG PS**	
0	0
1–2	1
≥ 3	2
**Sum of scores**	
Fit	0
Intermediate	1
Frail	≥ 2

*ECOG PS*, Eastern Cooperative Oncology Group performance status.

**Table 3:** Univariate and multivariate analyses of overall survival.

Characteristic			Univariate		Multivariate	
		N (%)	HR (95% CI)	*P*-value	HR (95% CI)	*P*-value
Age, years	< 75	569 (78.2)				
	≥ 75	159 (21.8)	1.65 (1.26–2.17)	< 0.001	1.79 (1.28–2.51)	0.001
CCI	< 3	620 (85.2)				
	≥ 3	108 (14.8)	2.55 (1.92–3.39)	< 0.001	2.89 (1.96–4.27)	< 0.001
ECOG PS	0	144 (19.8)				
	1–2	510 (70.1)	2.16 (1.44–3.23)	< 0.001	2.08 (1.19–3.64)	0.010
	≥ 3	74 (10.2)	3.72 (2.25–6.14)	< 0.001	3.18 (1.62–6.25)	0.001
Cytogenetics	Favorable	254 (65.0)				
	Unfavorable	137 (35.0)	1.24 (0.90–1.72)	< 0.001		
β_2_MG, mg/L	< 5.5	404 (57.8)				
	≥ 5.5	295 (42.2)	1.86 (1.45–2.39)	< 0.001	1.65 (1.15–2.37)	0.006
LDH	Normal	440 (74.8)				
	Abnormal	148 (25.2)	1.82 (1.36–2.43)	< 0.001	1.52 (1.06–2.19)	0.024
R-ISS	Stage I	40 (7.4)				
	Stage II	306 (56.4)	1.50 (0.76–3.00)	0.246	0.90 (0.44–1.83)	0.769
	Stage III	197 (36.3)	2.83 (1.42–5.63)	< 0.001	1.09 (0.49–2.42)	0.836

*OS*, overall survival; *HR*, hazard ratio; *95% CI*, 95% confidence interval; *CCI*, Charlson comorbidity index; *ECOG PS*, Eastern Cooperative Oncology Group performance status; *β*_*2*_*MG*, β_2_ microglobulin; *LDH*, lactate dehydrogenase; *R-ISS*, revised international staging system.

The differences in survival outcomes between frailty groups as identified using our frailty index were compared with those identified using the R-MCI and simplified frailty scale. The OS was significantly different between frailty groups as classified according to our frailty index (Table 4). There were also significant differences in survival outcomes between frailty groups as identified using the R-MCI and simplified frailty scale. Frail patients may have inferior survival outcomes compared with fit patients because they experience frequent and severe toxicities after the initiation of chemotherapy, and they should be administered reduced doses of chemotherapy or their treatment should be discontinued^4,17,19,20^. Therefore, accurate frailty assessment of elderly patients with MM can help to identify appropriate dosing schedules, manage toxicity, and improve survival outcomes. To estimate the feasibility of a frailty assessment tool, the differences in survival outcomes according to frailty have been considered important so far; however, the difference in the incidence rate of severe toxicity according to frailty is also important. However, clinical data regarding accurate toxicity profiles were not available in this study because of some limitations inherent to its retrospective design.

**Table 4:** Comparison of OS and EFS according to frailty models.

OS		N = 728			
Model	Group	N (%)	HR	(95% CI)	*P*-value
Our frailty scale	Fit	109 (15.0)			
	Intermediate	376 (51.6)	2.41	(1.43–4.06)	0.001
	Frail	243 (33.4)	4.61	(2.74–7.77)	< 0.001
R-MCI	Low	233 (32.0)			
	Intermediate	442 (60.7)	2.18	(1.59–2.98)	< 0.001
	High	53 (7.3)	3.28	(2.05–5.25)	< 0.001
Simplified frailty	Non-frail	303 (41.6)			
	Frail	425 (58.4)	1.67	(1.29–2.16)	< 0.001

EFS		N = 728			
Model	Group	N (%)	HR	(95% CI)	*P*-value
Our frailty scale	Fit	109 (15.0)			
	Intermediate	376 (51.6)	1.34	(0.92–1.96)	0.132
	Frail	243 (33.4)	2.06	(1.40–3.02)	< 0.001
R-MCI	Low	233 (32.0)			
	Intermediate	442 (60.7)	1.88	(1.42–2.40)	< 0.001
	High	53 (7.3)	3.12	(2.06–4.72)	< 0.001
Simplified frailty	Non-frail	303 (41.6)			
	Frail	425 (58.4)	1.77	(1.41–2.23)	< 0.001

*OS*, overall survival; *EFS*, event-free survival; *HR*,,hazard ratio; *95% CI*, 95% confidence interval; *R-MCI*, revised myeloma comorbidity index; *simplified frailty*, simplified frailty scale.

In conclusion, our new clinical frailty index, which is based on age, CCI, and ECOG PS, can easily assess frailty in elderly patients with MM and may be helpful in predicting survival outcomes. Further well-designed prospective studies are needed to confirm the association between frailty and incidence rates of toxicity and mortality.
